# A descriptive quantitative study of 7- and 8-year-old children’s outdoor recreation, cold exposure and symptoms in winter in Northern Finland

**DOI:** 10.1080/22423982.2017.1298883

**Published:** 2017-03-27

**Authors:** Hanna Rasi, Heli Kuivila, Tarja Pölkki, Risto Bloigu, Hannu Rintamäki, Marjo Tourula

**Affiliations:** ^a^ Nursing and Health Administration Science Research Unit, Oulu University, Oulu, Finland; ^b^ Nursing and Health Administration Science Research Unit, University of Oulu, Oulu, Finland; ^c^ Clinical Nursing Science, Oulu University Hospital, Children and Women, Oulu, Finland; ^d^ Medical Informatics and Statistics Research Group, University of Oulu, Aapistie, Oulu, Finland; ^e^ Finnish Institute of Occupational Health, Aapistie, Oulu, Finland; ^f^ Research Unit of Biomedicine, University of Oulu, Oulu, Finland; ^g^ Arctic Health, Faculty of Medicine and Thule Institute, University of Oulu, Aapistie, Oulu, Finland

**Keywords:** Outdoor, cold climate, children, cold exposure, well-being

## Abstract

**Background**: In Finland, children spend a lot of time outdoors in winter. Outdoor recreation in winter has a wide variety of effects on children’s well-being. Although children are a subgroup that is vulnerable to cold exposure, remarkably little research has been done on the subject.

**Objective:** The aim of this study was to describe children’s outdoor recreation, cold exposure and symptoms in winter in Northern Finland.

**Design:** This was a descriptive quantitative study. The participants consisted of 30 children aged 7–8 years who were living in the provinces of Lapland and Northern Ostrobothnia in Finland. Data were collected by using electronic data-logging thermometers fixed on children’s outerwear for a month. The thermometers recorded the environmental temperature every five minutes and from that temperature data, we were able to discern the exact amount and duration of children’s outdoor recreation. In addition, information on the children’s cold symptoms was collected with structured daily entries.

**Results:** Cold weather was not an obstacle to children’s outdoor activities in Finland. However, the duration of outdoor recreation shortened when the outdoor air temperature decreased. There were no significant differences between boys and girls in terms of time spent outdoors. Remarkably, every child reported symptoms associated with cold. Almost half of the children reported experiencing respiratory symptoms and some children also experienced cold pain and numbness.

**Conclusions:** The results of this study illustrate the many and varied effects that cold exposure can have on children’s health and well-being. In order to prevent negative health effects of cold exposure on children, structured prevention strategies are needed: therefore, children’s exposure to cold should be studied more. Future research should also bring out more the positive health effects of outdoor recreation on children’s growth and development.​​​​

## Background

In Finland children typically begin their outdoor lives at a very early age, when they start to take daytime naps outdoors.[1] By the 1920s, mothers were being instructed to have their children sleep outdoors in order to reduce infant mortality. Cold and fresh outdoor air was considered to be healthy and helpful in preventing illness in children.[2,3] Children’s outdoor sleeping is still a very common childcare practice in Finland.[4] Outdoor activities continue from early childhood to adulthood. Among Finnish adults, regularly 96% spend time outdoors, so outdoor recreation is common experience in Finland.[5] Also, Finns have a very strong bond with nature. Recurrent exercise in nature has been shown to increase emotional well-being.[6] In children, engaging in sufficient outdoor activities has been shown to have positive impacts including higher independent mobility, greater amounts of physical activity and lower prevalence of overweight.[7–9]

As a result of the geographical location of Finland at latitudes between 60º and 70º N, winters are long-lasting and cold. Winter begins when the mean ambient temperature stays permanently under 0°C and is the longest season in Finland.[10] Finland belongs to the very cold winter zone. Typically, the average temperature of the coldest month in Northern Finland is −15°C. The lowest recorded temperature in Finland was −51.5°C, measured in 1999.[10,11] General scientific understanding indicates that the climate is changing and that these changes increase the incidence and intensity of extreme weather events as well as their location and duration. Extreme weather phenomena are becoming more frequent.[10,12] It is suggested that, despite global warming, cold extremes in many regions will not be shorter or less severe than they have recently been.[13]

Cold exposure has a wide range of effects on human health. Connections between cold weather and increased mortality among children [14] and adults [15–17] have been shown in numerous studies. People in Finland are better-adapted to the cold than people, for example, in southern Europe;[18] however, in the current climate cold-related health problems in Finland are more significant than health problems caused by heat.[10] It has been reported that cold-related complaints are prevalent among the general population of Finland; most of the symptoms are reported to occur at −10°C to −20°C. Most general cold-related complaints are symptoms of the musculoskeletal or respiratory system, white finger and episodic symptoms in peripheral circulation.[19]

Matters that modify the effect of cold exposure are age, gender, general health, education, employment and physical activity.[20] Children are particularly vulnerable to extreme temperatures, including cold. Exposure to cold air has been associated with endocrine and respiratory diseases, intestinal infectious diseases and metabolic and nutritional diseases in children.[21] For example, cold spells have been connected to increases in childhood asthma symptoms.[22] For children with asthma, exposure of the face alone to cold temperatures may cause difficulties in breathing.[23] Especially during exercise in cold weather, also healthy Finnish people is reported to have respiratory symptoms, such as shortness of breath. [24] Children are at increased risk of frostbite.[25] Deep frostbite during childhood may cause long-term effects on health such as disturbing the development of the fingers.[26,27] Particular attention should be paid to children who have Raynaud’s syndrome, which causes susceptibility to frostbite.[28] Too little attention has been paid to children with neurological disorders and lower temperatures in the body’s extremities.[29] In summary, diverse health effects of winter outdoor living and cold exposure have been highlighted in previous studies; however, there is no previous research on cold exposure among 7- and 8-year-old Finnish children and its effects on children’s well-being and outdoor recreation. Therefore, the aim of this study was to describe children’s outdoor recreation, cold exposure and symptoms in winter in Northern Finland. In this study, by outdoor recreation we mean all the time children spend outdoors.

## Materials and methods

### Participants

A descriptive quantitative approach was adopted in this study because the purpose was to investigate the exact time that children spend outdoors. Thirty children (13 girls and 17 boys) aged 7–8 years were recruited from five different primary school in Finland. Children from this age group were selected to participate in the study because they are learning independent self-care, including clothing and protection against the cold. These children lived in Lapland and Northern Ostrobothnia in municipal centres (82%) and cities (18%). The distance of their trips to school ranged from 0.5 km to 4 km. Two of the children had asthma, two had a pollen allergy and one had atopic dermatitis. No other underlying diseases were reported.

### Data collection

Data were collected during a four-week period in January and February 2011 when outdoor air temperatures varied from −33.7°C to +3.4°C.[10] Smart button ACR temperature meters (ACR Systems Inc., ​​​​Surrey, Canada) were used to collect the data. The buttons were attached to each child’s outer clothing; they recorded environmental temperatures every five minutes throughout the study period; from January 24 to 20 February 2011.

After a month, the temperature data were downloaded into Excel files, so it was possible to discern the outdoor recreation and daily average time spent outdoors for each child. The data contained 3587 separate outdoor recreation periods, each of which included a number of variables such as temperature, time of day and duration. To ensure the correct temperatures were recorded, the thermometer data were compared with temperature data from the Finnish Meteorological Institute. While the thermometers recorded environmental temperatures, the children and their parents completed structured daily diary entries collecting information about the children’s daily condition, possible cold-related symptoms and the activities and hobbies in which they engaged while they were outdoors.

### Data analysis

The quantitative data were analysed using the Excel spreadsheet program and IBM SPSS Statistics 22.0 program.[30] By using statistical methods, the extensive data were aggregated by calculating each child’s daily average time spent outdoors. Frequencies were used to describe the data. Dependence on the variables was examined by using a scatter diagram. The incidence of cold symptoms was examined manually by collecting information from the children’s and parents’ diaries.

### Ethical considerations

Throughout the research process, the ethical principles of research were followed.[31,32] This type of research does not require permission from the local Ethics Committee according to Finnish law (2010/794, 2015/143). Research permission was obtained from the educational institutions through which the participants were recruited. Principals of five schools in northern Finland were contacted and asked for permission to approach first-grade pupils and their parents. Verbal and written information related to the study was given to all participants. The participants were informed about the voluntary nature of the study and notified that they could discontinue their participation in the study at any time. Because the participating children were underage, written consent for study was obtained from the children themselves and from their parents. The parents were given the researcher’s contact information for any questions. The data were maintained properly and participants’ anonymity was taken into account in the processing of the data.

## Results

### Children’s outdoor recreation

**Figure 1.**
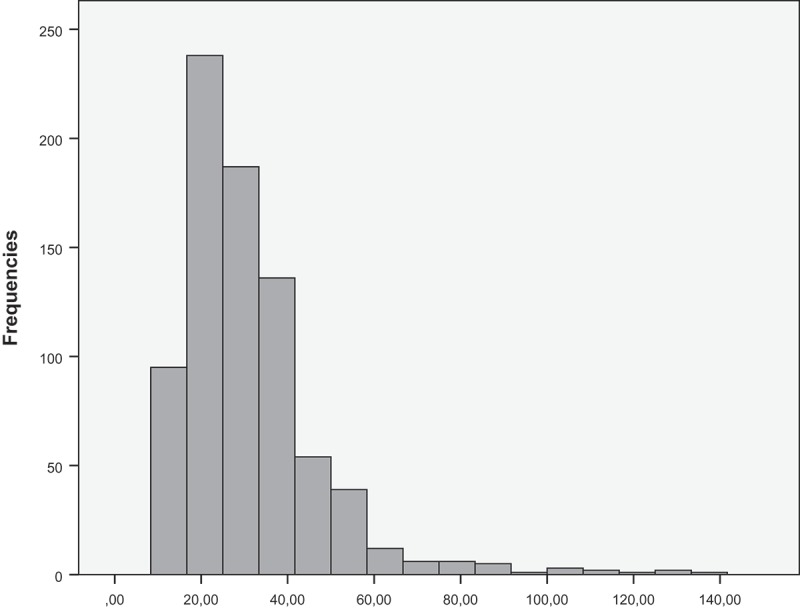
Duration of outdoor recreation. Only exposure times less than 150 min are presented.​​​​

The number of daily outdoor activities varied greatly among the children. There were no significant sex-based differences in children’s outdoor recreation (p = 0.899). The typical individual outdoor exposure time was 20–40 min (Figure 1); the maximal outdoor recreations lasted as long as 160, 215 and 270 min.

**Figure 2.**
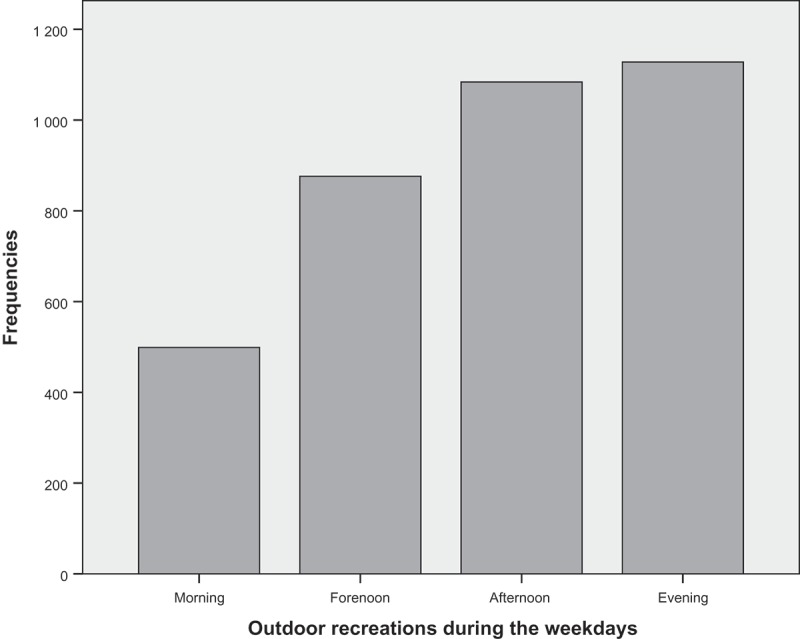
Outdoor recreations during the weekdays.

Outdoor recreation on weekdays was distributed throughout the day (Figure 2). During the school day, the children had mandatory outdoor activities due to planned breaks in the school schedule. Children also went outdoors in the evenings after school hours.

**Figure 3.**
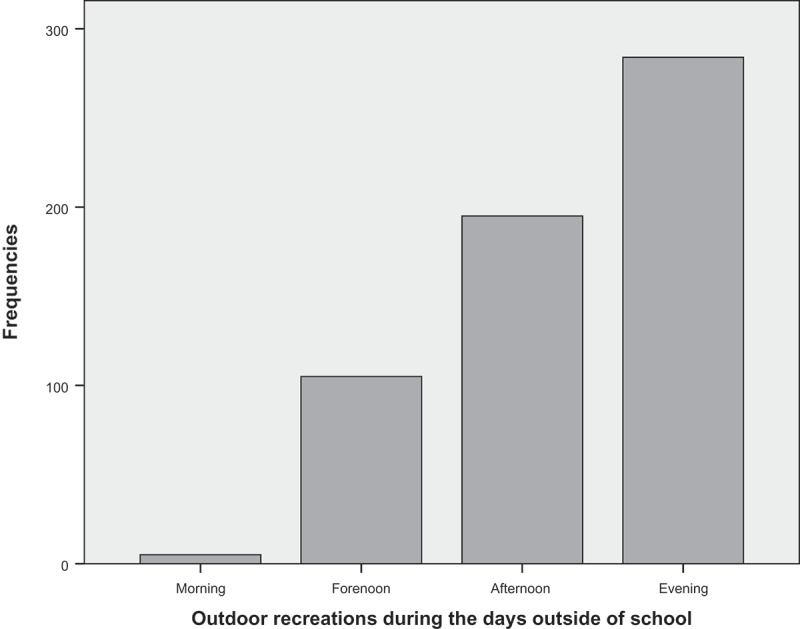
Outdoor recreations during the days outside of school.

Outdoor recreation during days outside of school occurred primarily during the afternoon and evening (Figure 3).

### Cold exposure

**Figure 4.**
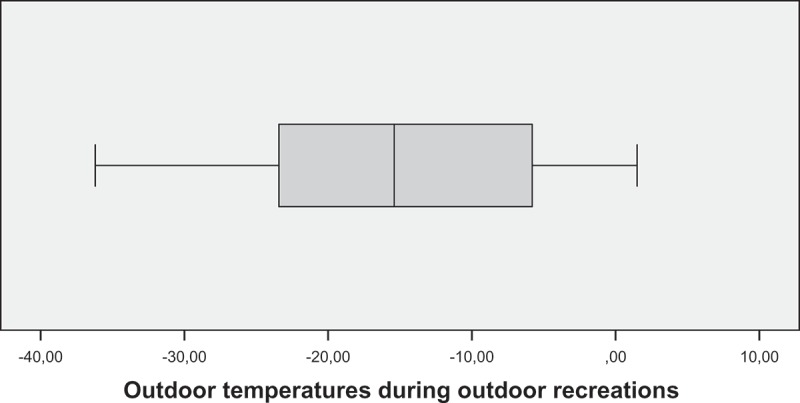
Outdoor temperatures during outdoor recreations.

During outdoor activities, children were exposed to significant variations in outdoor air temperatures (Figure 4). About half of the children’s outdoor activities took place in temperatures of −15°C or colder.

**Figure 5.**
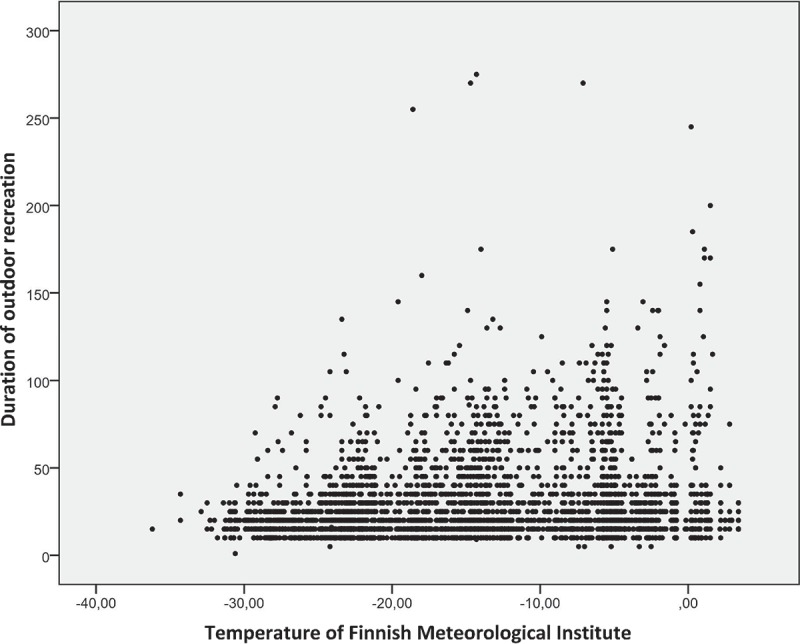
Temperature and duration of outdoor recreation.

**Figure 6.**
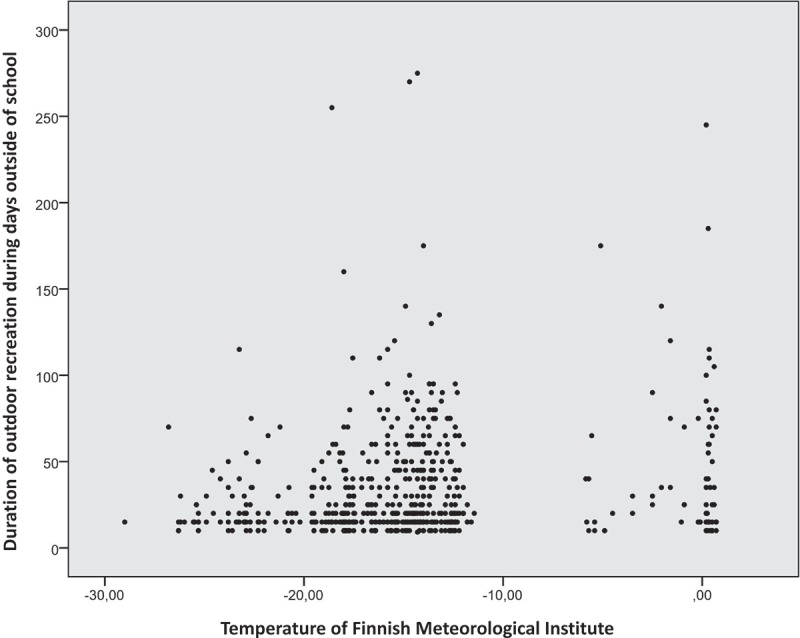
Temperature and duration of outdoor recreation during days outside of school.

The duration of the children’s outdoor recreational activities shortened when the outdoor air temperature decreased and lengthened when the outdoor air temperature increased (Figure 5). On days outside of school, outside air temperature affected the duration of the children’s outdoor recreation more than on weekdays (Figure 6).

### Cold associated symptoms and signs

Every child often reported having reddened cheeks. Additionally, four children reported occasional white areas on the cheeks. Four children occasionally felt cold, pain or numbness in the cheeks. Every child also reported redness of the nose during outdoor recreation. In addition, 12 children reported experiencing a runny nose in cold air. Almost half of the participants (14 children) reported redness in the earlobes and two of the children felt pain in the earlobes. Redness in fingers was reported by 12 children; 7 of those children felt pain and 2 of them felt numbness in their fingers. Ten of the study participants reported intermittent pain in the toes, and one of them felt numbness in the toes.

Almost half of the participants (14 children) reported some kind of respiratory symptoms connected to outdoor recreation and all of these children reported coughing. Mucous and respiratory distress were also reported. Twenty-three of the study participants often felt cold. Children were very physically active outdoors, and 22 of them reported sweating often. The children reported that they do not really stand in place while outdoors. Instead they reported engaging in many outdoor activities such as walking and running, cycling, skiing, ice skating, kick sledding, snowmobiling, downhill skiing, riding and swinging. In addition, the children liked very much playing in the snow, such as sliding down a hill or making a snowman or snow castle. Ploughing the snow was also reported.

## Discussion

The aim of this study was to describe children’s outdoor recreation, cold exposure and cold associated symptoms and signs in winter in Northern Finland. It has been shown that children’s outdoor recreation decreases during even relatively mild winters.[33] The results of this study showed that even very cold weather is not an obstacle to children’s outdoor life in Finland. However, colder weather shortened the duration of outdoor activities, especially during days outside of school. Unstructured outdoor play has been found to have a wide range of positive effects on children’s mental and physical well-being.[34] For health reasons children’s outdoor activities should be supported during the winter while also taking cold-related risks into account.

The key finding regarding cold-related disadvantages was that every child reported some symptoms associated with cold. A considerable number of the children experienced warning signals of approaching frostbite such as pain and numbness in the extremities. It is important to teach children to recognise pre-frostbite marks and make decisions to protect their health. The experience of pain and numbness requires immediate action and increased clothing.[35] The majority of children often felt cold, so the results show that 7- and 8-year-old children still need the active support of parents and caregivers to select appropriate and adequate clothing in cold weather. In the school environment, teachers and other adults should pay attention to children’s clothing. Schools should also provide space for children to change clothing and dry clothes that have got wet.

Worldwide there is an ongoing refugee crisis – the largest such crisis since the Second World War. The number of asylum seekers in Finland has grown rapidly in recent years. In 2015, 32,476 asylum-seekers arrived in Finland.[36] There are significant differences between different countries’ populations in the development of skills and knowledge related to protection against the cold. In cold countries people are used to adjusting their behaviour in accordance with the requirements of the cold air. In the south, people do not recognise health problems related to the cold in the same way as people in the north.[18] Particular attention should be paid to supporting immigrant children and their parents in learning skills to cope with the cold.

This study strength is that, to the best of our knowledge, this is the ﬁrst study which speciﬁcally investigates primary-school pupils’ outdoor recreation and cold exposure in Northern Finland. By using an innovative data collection method, we managed to collect legitimate, interesting and unique research data. One strength of this study was also our use of electronic thermometers in data collection; otherwise it would have been difficult for participants to report accurate temperature and times they spent outdoors and exposed to cold. In order to increase the reliability of the research, the diary was reviewed by an expert researcher of cold exposure and pre-tested on five parents before the collection of the data.

This study also has limitations. Volume of cold exposure varies greatly during the winter and from year to year. The average of temperature of the four-week period was colder than normal,[10] so these data represent only one example of cold winter period in Finland. The study population was small, so the results cannot be directly generalised. In addition, the study was carried out only in the north of Finland where children are accustomed to and adapted to cold winter conditions. In the future, it would be useful to study the same research questions in southern Finland where children’s behaviours in cold weather may differ. Research should also be extended internationally.

## Conclusions

The novelty of this research is that it increases our knowledge of how cold exposure can influence children’s outdoor recreation and well-being. While health precautions against hot weather have been a matter of worldwide interest, it is also important to pay attention the harmful effects of cold exposure, especially for children.

This study’s results can be utilised to promote children’s health and well-being and in the development of cold management strategies in northern habitats. Substantial prevention strategies are needed. The results of the study support the addition of knowledge of cold exposure to the primary school curriculum and integration programs for immigrants. It would be important to study the experiences of refugee children in adapting to the cold climate. Additional research should focus on the health impacts of cold exposure for children. That kind of research data is needed as a basis for the development of cold-management strategies both nationally and internationally. On a methodological level, this study showed the usability of physiological measurement methods in health science research. The development of new, innovative research methods should be further investigated.
